# Multimorbidity Accumulation by Race or Ethnicity and Body-Weight Status Among Middle-Aged and Older Americans

**DOI:** 10.1093/geroni/igaa057.822

**Published:** 2020-12-16

**Authors:** Anda Botoseneanu, Sheila Markwardt, Heather Allore, Corey Nagel, Jason Newsom, David Dorr, Ana Quinones

**Affiliations:** 1 University of Michigan, Dearborn, Michigan, United States; 2 School of Public Health - Portland State University, Portland, Oregon, United States; 3 Yale University, New Haven, Connecticut, United States; 4 University of Arkansas for Medical Sciences, Little Rock, Arkansas, United States; 5 Portland State University, Portland, Oregon, United States; 6 Oregon Health & Science University, Portland, Oregon, United States

## Abstract

Obesity and multimorbidity are more prevalent among U.S. racial/ethnic minority groups. Evaluating racial/ethnic disparities in multimorbidity accumulation according to body-mass index (BMI) may guide interventions to reduce multimorbidity burden in vulnerable racial/ethnic groups. Data from the 1998-2016 Health & Retirement Study (N=8,106, 51-55 years at baseline) and generalized estimating equations models with inverse probability weights estimated the accumulation of seven chronic diseases (arthritis, cancer, diabetes, heart disease, hypertension, lung disease, and stroke) between racial/ethnic groups [Non-Hispanic White (reference; 64.2%), Non-Hispanic Black (21.6%), Hispanic (14.2%)]. Overweight and obesity were more prevalent in Black (82.3%) and Hispanic (78.9%) than White (70.9 %) participants at baseline. Initial burden of morbidity was higher among Black participants [risk ratio (RR) =1.3, p<0.001] but similar among Hispanic compared with White participants; and higher in overweight or greater BMI categories compared with normal BMI (RR=1.07, 1.15, 1.22, p<0.001, for overweight, obese 1, and obese 2/3 BMI, respectively). Disease accumulation did not differ among racial/ethnic groups. Higher BMI was associated with less disease accumulation compared with the normal BMI category (RR=0.99, 0.98, 0.97, all p<0.001, for overweight, obese 1, and obese 2/3 BMI, respectively, per two-year interval). Black participants crossed the threshold of multimorbidity (≥2 diseases) 4-6 years earlier than White and Hispanic participants. There are substantial differences in initial disease burden between Black and White middle-aged/older adults, but not in the accumulation of disease, suggesting the need to intervene prior to entering middle age to reduce disparities in the burden of multimorbidity among vulnerable racial minorities.

